# A novel candidate species of *Anaplasma* that infects avian erythrocytes

**DOI:** 10.1186/s13071-018-3089-9

**Published:** 2018-09-24

**Authors:** Ralph Eric Thijl Vanstreels, Michael J. Yabsley, Nola J. Parsons, Liandrie Swanepoel, Pierre A. Pistorius

**Affiliations:** 1 Marine Apex Predator Research Unit (MAPRU), Institute for Coastal and Marine Research, Nelson Mandela University, Port Elizabeth, South Africa; 2 DST/NRF Centre of Excellence at the Percy FitzPatrick Institute for African Ornithology, Department of Zoology, Nelson Mandela University, Port Elizabeth, South Africa; 3 Warnell School of Forestry and Natural Resources, The University of Georgia, Athens, GA USA; 4 Southeastern Cooperative Wildlife Disease Study, Department of Population Health, College of Veterinary Medicine, The University of Georgia, Athens, GA USA; 5 Southern African Foundation for the Conservation of Coastal Birds (SANCCOB), Cape Town, South Africa

**Keywords:** “*Candidatus* Anaplasma sphenisci”, African penguin (*Spheniscus demersus*), Avian erythrocytes, South Africa, Phylogeny, *16S* rRNA and *groEL* genes

## Abstract

**Background:**

*Anaplasma* spp. are Gram-negative obligate intracellular bacteria transmitted by ticks. Even though numerous studies have detected DNA from *Anaplasma* spp. in the blood of birds, thus far mammals were the only vertebrates demonstrated to serve as competent hosts to these organisms. We report a novel candidate species of *Anasplasma* that was associated with cytoplasmic inclusions in the erythrocytes of an African penguin (*Spheniscus demersus*) in South Africa.

**Methods:**

Cytoplasmic inclusions were morphologically characterized from freshly-produced blood smears, and phylogenetic analysis of *16S rRNA* and *groEL* genes were used to evaluate the evolutionary relationships of the organism to other *Anaplasmataceae*.

**Results:**

Dark-purple round or oval inclusions consistent with *Anaplasmataceae* morulae were observed in the cytoplasm of erythrocytes. Phylogenetic trees produced using different methods agreed that the organism detected in this study belongs to the genus *Anaplasma*, and suggested that it is most closely related to the cluster comprising *A. centrale*, *A. capra*, *A. marginale* and *A. ovis*. We propose provisionally naming the strain detected in this study as “*Candidatus* Anaplasma sphenisci”.

**Conclusions:**

This is the first species of *Anaplasma* shown to produce cytoplasmic inclusions in avian cells, opening the possibility that cytoplasmic inclusions in avian erythrocytes that had previously been attributed to *Aegyptianella* sp. might in fact correspond to *Anaplasma*. Further studies on the molecular biology of avian-infecting *Anaplasmataceae* will be valuable to provide insight into the evolution and epidemiology of these organisms.

**Electronic supplementary material:**

The online version of this article (10.1186/s13071-018-3089-9) contains supplementary material, which is available to authorized users.

## Background

*Anaplasmataceae* (Alphaproteobacteria: Rickettsiales) are Gram-negative obligate intracellular bacteria found exclusively within membrane-bound inclusions or vacuoles in the cytoplasm of vertebrate and invertebrate host cells [1]. This family comprises five recognized genera (*Aegyptianella*, *Anaplasma*, *Ehrlichia*, *Neorickettsia* and *Wolbachia*) [1], and four candidate genera (“*Candidatus* Cryptoplasma”, “*Candidatus* Neoehrlichia”, “*Candidatus* Xenohaliotis”, and “*Candidatus* Xenolissoclinum”) [2–5].

The genus *Anaplasma* currently includes nine species, six candidate species as well as numerous unclassified species, all of which are either known or believed to be tick-borne (Table 1). Depending on the involved species, these organisms infect the cytoplasm of blood cells (erythrocytes, leukocytes or platelets), bone marrow precursor cells, or endothelial cells of vertebrates, forming pleomorphic clusters of bacteria (morulae) [6]. Mammals are the only vertebrates demonstrated thus far to be competent hosts of *Anaplasma* spp., but numerous studies have detected DNA from *Anaplasma* spp. (especially *A. phagocytophilum*) in the blood of birds and in the tissues of ticks collected from birds [7–11]. However, no studies demonstrated the presence of *Anaplasma* spp. cytoplasmic inclusions within blood cells of birds, and it was therefore considered unclear whether these organisms are able to infect avian cells or merely remain viable in the avian plasma [10, 12].

**Table 1:** Overview of the species and candidate species of the genus *Anaplasma* [1, 41–54]

Species	Tick host	Vertebrate host	Host cells
*Anaplasma bovis*	*Haemaphysalis*, *Rhipicephalus*, *Amblyomma*	Domestic and wild ruminants, small mammals	Monocytes
*Anaplasma capra*	*Haemaphysalis*	Domestic and wild ruminants, humans	Not known
*Anaplasma caudatum*	Not known	Domestic and wild ruminants	Erythrocytes
*Anaplasma centrale*	*Ixodes*, *Haemaphysalis*	Domestic and wild ruminants	Erythrocytes
*Anaplasma marginale*	*Ixodes*, *Dermacentor*	Domestic ruminants	Erythrocytes
*Anaplasma odocoilei*	Not known	Wild ruminants	Platelets
*Anaplasma ovis*	*Dermacentor, Hyalomma, Rhipicephalus*	Domestic and wild ruminants, humans	Erythrocytes
*Anaplasma phagocytophilum*	*Ixodes*, *Dermacentor, Hyalomma, Rhipicephalus*	Domestic and wild ruminants, horses, dogs, cats, rabbits, rodents, insectivores, wild swine, humans	Granulocytes
*Anaplasma platys*	*Rhipicephalus*	Dogs, camels	Platelets
“*Candidatus* Anaplasma boleense”	*Hyalomma*	Not known	Not known
“*Candidatus* Anaplasma camelii”	Not known	Camels	Not known
“*Candidatus* Anaplasma corsicanum”	Not known	Domestic ruminants	Not known
“*Candidatus* Anaplasma ivorensis”	*Amblyomma*	Not known	Not known
“*Candidatus* Anaplasma mediterraneum”	Not known	Domestic ruminants	Not known
“*Candidatus* Anaplasma rodmosense”	Not known	Rats	Not known
“*Candidatus* Anaplasma sphenisci”^a^	Not known	African penguins	Erythrocytes

^a^Proposed in this study

On the other hand, cytoplasmic inclusions observed in the erythrocytes of birds have been traditionally attributed to members of the genus *Aegyptianella*. Currently the only recognized species of *Aegyptianella* is the avian-infecting *Aegyptianella pullorum* [13, 14], and the validity of other proposed *Aegyptianella* spp. remains unclear and the genus has been considered *incertae sedis* [1, 13, 15]. *Aegyptianella pullorum* infects the cytoplasm of erythrocytes forming pleomorphic inclusions with a diameter ranging between 0.3–4.0 μm, and has been demonstrated to infect chickens, turkeys, ducks, geese and quails [13, 16, 17]. A previous genetic study revealed that *Ae. pullorum* from turkeys is closely related to *Anaplasma* [17], leading some authors to suggest that *Ae. pullorum* should be reclassified as an *Anaplasma* [18, 19], but currently there is no consensus on this suggestion [20].

In this study, we describe a novel candidate species of *Anaplasma* that is associated with cytoplasmic inclusions in the erythrocytes of the African penguin (*Spheniscus demersus*), and discuss the phylogenetic relationships of this organism to other *Anaplasmataceae*.

## Methods

The Southern African Foundation for the Conservation of Coastal Birds (SANCCOB) facility in Cape Town (33°50'02"S 18°29'29"E) receives and rehabilitates oiled, sick, and injured marine and coastal birds along the coast of South Africa. Cytoplasmic inclusions consistent with *Anasplasmataceae* were observed in the erythrocytes of an adult African penguin during the examination of blood smears as a part of routine veterinary checks. The individual history of the studied penguin is summarized in Additional file 1.

Blood was obtained from the tarsal vein and thin blood smears were freshly prepared, fixed and stained with a modified Wright-Giemsa stain (Kyro-Quick, Kyron Laboratories, Benrose, South Africa). The percentage of erythrocytes with inclusions was estimated with manual counts of erythrocytic inclusions and software-assisted counts of *c.*2000 erythrocytes; erythrocytes were counted from photographs of 20 randomly-selected microscope fields under 1000× magnification using ImageJ 1.46r [21, 22]. ImageJ 1.46r was also used to measure the width of cytoplasmic inclusions. The following morphological characteristics were recorded for 100 erythrocytic inclusions: position (polar, subpolar, median), contact with host cell margins (contact with outer margin, contact with nuclear margin, no contact with margins), and the presence of adjacent indentation of host cell outer margin (present, absent).

DNA was extracted from frozen blood using the DNeasy Blood and Tissue kit (Qiagen, Hilden, Germany) following the manufacturer’s instructions. A 927 bp segment of the *16S rRNA* gene was amplified using the primers 8F and 1492R [23]. A 939 bp segment of the *groEL* gene was amplified using a nested PCR with the primary primers HS1 and HS6 and secondary primers HS43 and HSVR [24, 25]. Amplification products were sequenced using Sanger bidirectional sequencing.

MegaBLAST [26] was used to identify publicly-available sequences that were highly similar to those obtained in this study. Phylogenetic analyses of the *16S rRNA* and *groEL* genes were conducted to compare sequences obtained in this study to publicly-available sequences of *Anaplasmataceae* (Additional file 2: Table S1). Sequences were aligned using ClustalW [27] as implemented in MEGA 7 [28]. General Time Reversible model with invariant sites and gamma distribution (GTR+I+G) was used for both genes as recommended by jModelTest 2.1.10 [29]. Neighbor-Joining trees (maximum composite likelihood, including transitions and transversions) and Maximum Likelihood trees (nearest neighbor interchange) were produced using MEGA 7; bootstrap values were calculated from 5000 replicates. Bayesian trees (two simultaneous Markov chains, 5 million generations, sampling every 1000 generations) were produced using MrBayes 3.2.6 [30]; posterior probabilities were calculated after discarding the first 25% trees as a ‘burn-in’ step. Phylogenetic analyses were conducted separately for each gene and also for concatenated (Neighbor-Joining and Maximum Likelihood) or partitioned (Bayesian) sequences of the two genes.

## Results

**Fig. 1:**
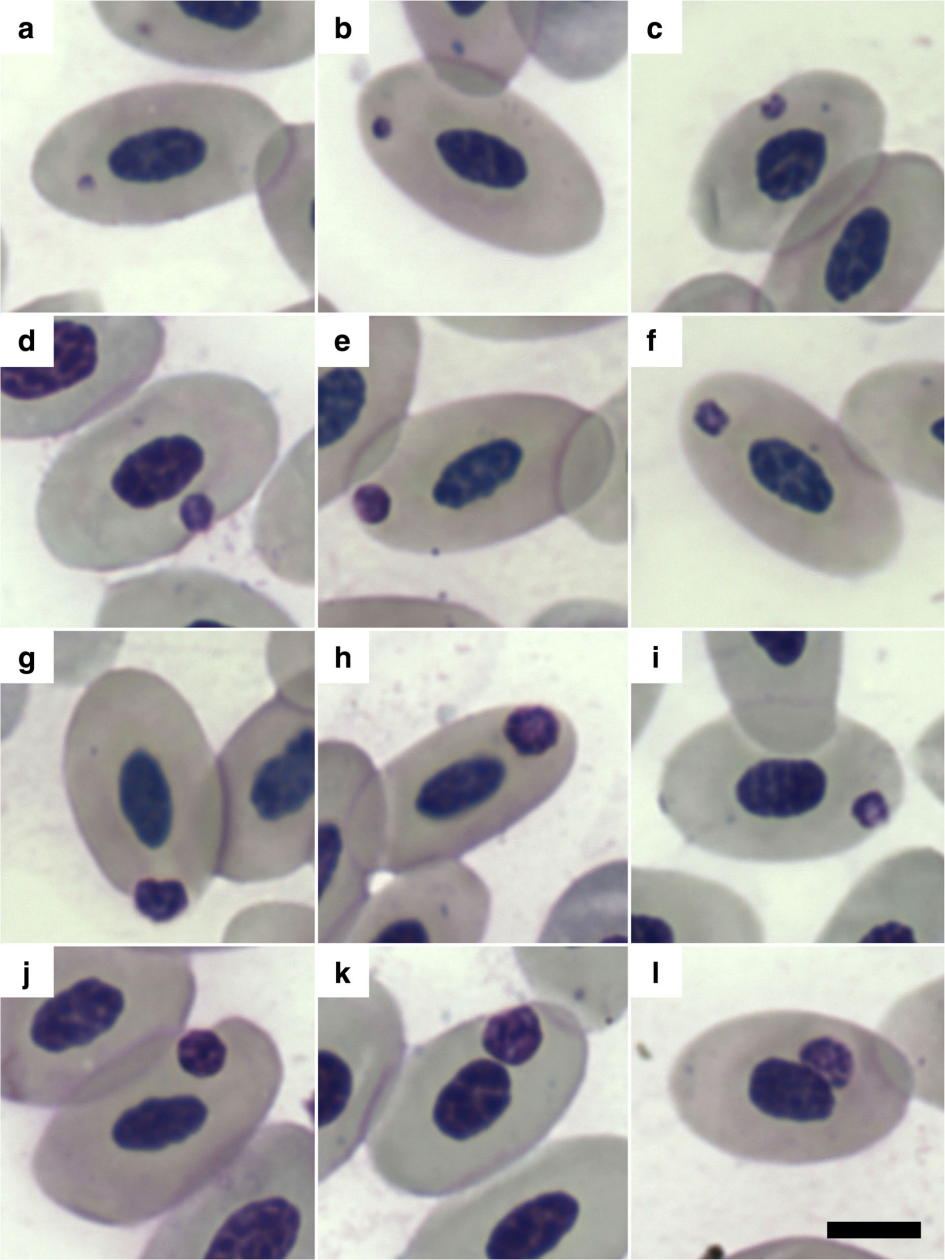
Cytoplasmic inclusions attributed to “*Candidatus* Anaplasma sphenisci” in the erythrocytes of an African penguin (*Spheniscus demersus*). Modified Wright-Giemsa stain. Inclusions ranged in size from small dots (**a, b**) to pleomorphic structures that were smaller than the host cell nucleus (**c-l**) and were found at a polar (**a**, **e**-**h**), subpolar (**b, i-l**) or median position (**c, d**), at times touching the host cell outer margins (**c-e, g-k**) or the host cell nucleus (**l**). *Scale-bar*: 5 μm

Dark-purple round or oval inclusions consistent with *Anaplasmataceae* morulae were observed in the cytoplasm of 0.10% of the erythrocytes (Fig. 1). In most cases these inclusions had a dense and homogeneous texture with a slightly paler center (e.g. Fig. 1f), but in some cases it was possible to identify irregularly distributed dense dots or crescent-shaped areas (e.g. Fig 1j and 1l). The inclusions (*n* = 100) had a width of 2.28 ± 0.56 μm (range: 1.02–3.33 μm), and were positioned as follows: 46% polar (e.g. Fig. 1g), 39% subpolar (e.g. Fig. 1k), and 15% median (e.g. Fig. 1d). Most of the inclusions (75%) appeared to be in contact with the outer margins of the host cell (e.g. Fig. 1j), 7% appeared to be in contact with the host cell nucleus (e.g. Fig. 1l), and 18% did not appear to be in direct contact with any host cell margins (e.g. Fig. 1e). The outer margins of the host cell were deformed and a small indentation was visible in 63% of the erythrocytes where inclusions were in direct contact with the host cell outer margin (e.g. Fig. 1d and i). In addition to the erythrocytic inclusions, one lymphocyte contained dark-purple cytoplasmic inclusions (Additional file 3: Figure S1); however, despite extensive searching (> 1000 leukocytes examined), no other leukocytes or thrombocytes were seen with similar cytoplasmic inclusions and it was therefore not possible to determine the identity of these structures.

**Fig. 2:**
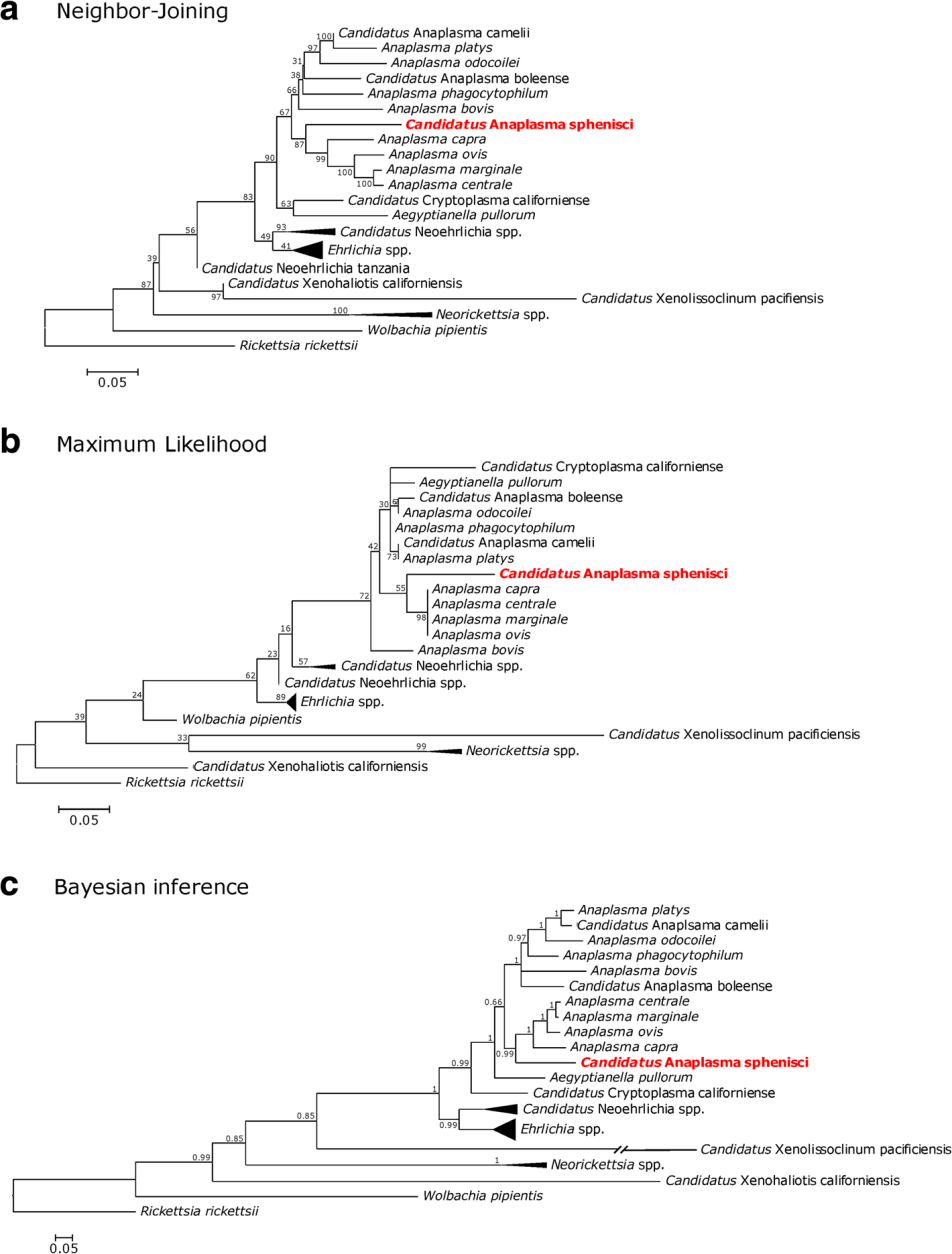
Phylogenetic relationships of “*Candidatus* Anaplasma sphenisci” as determined by different phylogenetic methods based on partial sequences of the *16S rRNA* and *groEL* genes. Branch lengths are drawn proportionally to evolutionary distance (scale-bars are shown). Numbers adjacent to nodes indicate bootstrap values (**a, b**) or posterior probabilities (**c**)

Molecular detection of *16S rRNA* and *groEL* sequences confirmed the presence of an organism belonging to *Anaplasmataceae*. MegaBLAST found that the closest publicly-available sequences were *A. marginale* (Genbank KU686794) with 96.8% sequence identity for the *16S rRNA* gene and *A. phagocytophilum* (CP015376) with 78.7% sequence identity for the *groEL* gene. Relative to publicly-available sequences of *A. pullorum*, sequence identity was 89.1% for the *16S rRNA* gene and 76.3% for the *groEL* gene. Phylogenetic trees of the *16S rRNA* and *groEL* sequences differed in relation to the topology (Fig. 2, Additional file 4: Figure S2), but the different phylogenetic methods agreed that the organism detected in this study belongs to the genus *Anaplasma*, and that it is most closely related to the cluster comprising *A. centrale*, *A. capra*, *A. marginale* and *A. ovis*.

## Discussion

Considering the host species and the phylogenetic relationship to other *Anaplasma* species, we propose provisionally naming the bacterial strain detected in this study as “*Candidatus* Anaplasma sphenisci” (derived from Spheniscidae, the family of the penguin host).

The finding that mammals are not the only vertebrate hosts of *Anaplasma* advances the question of whether *Aegyptianella pullorum* should be reclassified as *Anaplasma pullorum*. Our phylogenetic analyses agree that *Ae. pullorum*, *Anaplasma* spp. and “*Candidatus* Cryptoplasma californiense” are monophyletic; however, different phylogenetic methods disagree on the relationships amongst these groups (see Fig. 2 and Additional file 4: Figure S2). In the absence of additional information on the genetic diversity of other avian-infecting *Anaplasmataceae*, the question whether the reclassification of *Ae. pullorum* is warranted remains unresolved.

The fact that *Anaplasmataceae*-like cytoplasmic inclusions have also been recorded in the erythrocytes of numerous other avian species (e.g. doves, cranes, kites, pheasants, psittacines and passerines) [13, 31–33] suggests that other species of avian-infecting *Anaplasmataceae* may exist but have yet to be described. This seems particularly plausible in the case of the intraerythrocytic inclusions originally described as “*Aegyptianella botuliformis*” [31] and “*Aegyptianella minutus*” [32], both of which produce erythrocytic inclusions that are morphologically distinct from those traditionally attributed to *Ae. pullorum*. Similarly, the Rickettsiales-like cytoplasmic inclusions observed in the erythrocytes of a king penguin (*Aptenodytes patagonicus*) that died while in care at SANCCOB [34] appeared distinct from those observed in this study (smaller, finer structure, with pale central vacuoles, more clearly-defined dark purple dots, did not distort the outer margin of the host cell) and likely also represent a distinct (and potentially novel) organism. Future studies on the molecular biology of avian-infecting *Anaplasmataceae* will therefore be valuable to provide insight into the evolution of these organisms and indicate the most appropriate nomenclature for *Aegyptianella*.

Over the past few decades, tens of thousands of blood smears from African penguins have been examined at SANCCOB. However, because “*Candidatus* Anaplasma sphenisci” was not known to exist, it is possible that its inclusions were mistakenly interpreted as corresponding to small round forms of *Babesia* spp., degenerative changes, or staining artifacts. Our results therefore do not necessarily indicate that this is a novel or emerging pathogen, and further studies will be necessary to evaluate its prevalence in African penguins.

The vectors of “*Candidatus* Anaplasma sphenisci” are not known. The soft tick *Ornithodoros capensis* is a common parasite of African penguins, including in South Africa [35], and is thus the most probable vector. However, the hard tick *Ixodes uriae* is also thought to occur on the coast of South Africa and, even though it has not yet been recorded on African penguins, it is a frequent parasite of other penguin species elsewhere [36]. Both *O. capensis* and *I. uriae* are shared by a large number of seabird species [37], including seabirds that breed sympatrically with African penguins such as Bank and Cape cormorants (*Phalacrocorax neglectus* and *Phalacrocorax capensis*), Cape gannets (*Morus capensis*) and Kelp gulls (*Larus dominicanus*) [38–40]. The potential therefore exists for the transmission of this bacterium to other seabird species.

## Conclusions

“*Candidatus* Anaplasma sphenisci” is the first species candidate of *Anaplasma* shown to produce cytoplasmic inclusions in avian cells. This opens the possibility that cytoplasmic inclusions in avian erythrocytes that had previously been attributed to *Aegyptianella* sp. might in fact correspond to *Anaplasma*. It is therefore clear that the diversity and host range of *Anaplasma* spp. might have been underestimated, and further studies on the molecular biology of avian-infecting *Anaplasmataceae* will be valuable to provide insight into the evolution and epidemiology of these organisms.

## Additional files

Additional file 1: Text. Individual history of the studied African penguin. (PDF 25 kb)

Additional file 2: **Table S1.** GenBank accession codes for the sequences analyzed. (PDF 30 kb)

Additional file 3: **Figure S1.** Cytoplasmic inclusions in a lymphocyte of an African penguin (*Spheniscus demersus*) infected by “*Candidatus* Anaplasma sphenisci”. The lymphocyte with cytoplasmic inclusions (upper right) and a normal lymphocyte (lower left) are shown. Cytoplasmic inclusions had a width of 1.98 ± 0.54 μm (range = 0.86–3.10 μm). Modified Wright-Giemsa stain. *Scale-bar*: 5 μm. (PDF 8025 kb)

Additional file 4: **Figure S2.** Phylogenetic relationships of “*Candidatus* Anaplasma sphenisci” as determined by different phylogenetic methods based on partial sequences of the *16S rRNA* and *groEL* genes. Branch lengths are drawn proportionally to evolutionary distance (scale-bars are shown). Numbers adjacent to nodes indicate bootstrap values (a, b, d, e) or posterior probabilities (c, g). (PDF 110 kb)

## Abbreviations

PCR: polymerase chain reaction
SANCCOB: Southern African Foundation for the Conservation of Coastal Birds
