# Supplementary material for: A novel candidate species of Anaplasma that infects avian erythrocytes
Source: Parasit Vectors. 2018 Sep 24;11:525. doi: 10.1186/s13071-018-3089-9 (PMC6154407; doi:10.1186/s13071-018-3089-9)
Supplement: Supplementary file 1 — Text. Individual history of the studied African penguin. (PDF 25 kb) [file 13071_2018_3089_MOESM1_ESM.pdf]

Additional file 1: Text. Individual history of the studied African penguin.

On 01-Sep-2016, an adult African penguin with a leg injury was rescued from the Stony Point colony (34°22’26”S 18°53’41”E), situated approximately 50 kilometers south-east of Cape Town. The penguin was transported to SANCCOB’s facility in Cape Town (33°50’02”S 18°29’29”E), where it received the individual identification “AP450-2016”. Upon admission, the bird was in good body condition (body mass 2.96 kg) with a packed cell volume (PCV) of 30%. As part of the treatment for its leg injury, the bird was treated with antimicrobials (clindamycin 100 mg/kg q24h PO) for 28 days starting on the day of admission. The PCV gradually improved in the weeks following admission, reaching a maximum of 49% on 10-Oct-2016, after which it decreased to 39% on 17-Oct-2016, 35% on 31-Oct-2016, and 33% on 07-Nov-2016.

Examination of blood smears produced on 31-Oct-2016 and 07-Nov-2016 revealed the presence of unusual structures within the cytoplasm of the erythrocytes. These structures were considered distinct from those of protozoa that are known to infect the erythrocytes African penguins (*Babesia peircei*, *Plasmodium* spp.). Stained blood smears and a frozen blood sample produced on 07-Nov-2016 were stored for later analysis, and the photographs and DNA sequences analyzed in this study were derived from those samples. On 07-Nov-2016, the differential leukocyte count indicated a relative heterophilia (72% heterophils, 24.5% lymphocytes, 2% eosinophils, 1.5% monocytes), and a mild erythrocytic regenerative response was present (20.6% of erythrocytes corresponding to young cells).

On 14-Nov-2016, the PCV had further decreased to 26%, and empirical treatment with amoxicillin and clavulanic acid (respectively 25 mg/kg and 6.25 mg/kg, q12h PO) was initiated for 10 days. The PCV stabilized at 24% on 22-Nov-2016, after which the bird gradually improved, with the PCV increasing to 36% by 30-Jan-2017. In February 2017, after molting and with the leg injury completely healed, the bird passed the release criteria (apparently healthy, good body condition, waterproof plumage, normal behavior and swimming, wariness of predators and humans) and was released at Stony Point on 08-Feb-2017.
