# Supplementary material for: A novel candidate species of Anaplasma that infects avian erythrocytes
Source: Parasit Vectors. 2018 Sep 24;11:525. doi: 10.1186/s13071-018-3089-9 (PMC6154407; doi:10.1186/s13071-018-3089-9)
Supplement: Supplementary file 2 — Table S1. GenBank accession codes for the sequences analyzed. (PDF 30 kb) [file 13071_2018_3089_MOESM2_ESM.pdf]

Additional file 2: Table S1. GenBank accession codes for the sequences analyzed.

**Species**	***16S rRNA***	***groEL***
Aegyptianella pullorum	AY125087	AY150648
Anaplasma bovis	KY425420	KY425421
Anaplasma capra	KM206273	KM206275
Anaplasma centrale	CP001759	CP001759
Anaplasma marginale	CP006846	CP006846
Anaplasma odocoilei	JX876644	JX876642
Anaplasma ovis	AF414870	AF441131
Anaplasma phagocytophilum	CP015376	CP015376
Anaplasma platys	AY077619	AY077621
"*Candidatus* Anaplasma boleense"	KX987334	KX987391
"*Candidatus* Anaplasma camelii"	KF843823	KJ814955
"*Candidatus* Anaplasma sphenisci"	MG748724	MG748859
"*Candidatus* Cryptoplasma californiense"	KP276585	KP276600
Ehrlichia canis	NC_007354	NC_007354
Ehrlichia chaffeensis	NZ_CP007476	NZ_CP007476
Ehrlichia ewingii	U96436	AF195273
Ehrlichia mineirensis	JX629805	JX629806
Ehrlichia muris	NC_023063	NC_023063
Ehrlichia occidentalis *	not available	KY425507
Ehrlichia ovina *	AF318946	not available
Ehrlichia ruminantium	NC_005295.2	NC_005295.2
"*Candidatus* Ehrlichia khabarensis"	KR063138	KR063139
"*Candidatus* Ehrlichia occidentalis"	not available	KY425507
"*Candidatus* Ehrlichia ornithorhynchi"	MF069159	MF069155
"*Candidatus* Ehrlichia ovata"	not available	DQ672553
"*Candidatus* Ehrlichia regneryi"	KF843826	KJ814961
"*Candidatus* Ehrlichia shimanensis"	AB074459	AB074462
"*Candidatus* Ehrlichia urmitei"	not available	KU212375
"*Candidatus* Neoehrlichia arcana"	KU865441	KU865443
"*Candidatus* Neoehrlichia australis"	KU865414	KU865345
"*Candidatus* Neoehrlichia chilensis" *	not available	MF805779
"*Candidatus* Neoehrlichia lotoris"	NZ_LANX01000001	NZ_LANX01000001
"*Candidatus* Neoehrlichia mikurensis"	AB084582	AB084583
"*Candidatus* Neoehrlichia tanzania"	KT895260	not available
Neorickettsia helminthoeca	NZ_CP007481	NZ_CP007481
Neorickettsia risticii	NC_013009	NC_013009
Neorickettsia sennetsu	NC_007798	NC_007798
Rickettsia rickettsia	CP003311	CP003311
Wolbachia pipientis	JX182384	NWVJ01000010
"*Candidatus* Xenohaliotis californiensis"	AF133090.2	not available
"*Candidatus* Xenolissoclinum pacificiensis"	AXCJ01000001	AXCJ01000003

Note: * = Not included in the phylogenetic analyses with concatenated sequences.
