# Supplementary material for: A novel candidate species of Anaplasma that infects avian erythrocytes
Source: Parasit Vectors. 2018 Sep 24;11:525. doi: 10.1186/s13071-018-3089-9 (PMC6154407; doi:10.1186/s13071-018-3089-9)

Additional file 3: Figure S1. Cytoplasmic inclusions in a lymphocyte of an African penguin (*Spheniscus demersus*) infected by "*Candidatus* Anaplasma sphenisci". The lymphocyte with cytoplasmic inclusions (upper right) and a normal lymphocyte (lower left) are shown. Cytoplasmic inclusions had a width of 1.98 ± 0.54 μm (range = 0.86–3.10 μm). Modified Wright-Giemsa stain. *Scale-bar*: 5 μm.

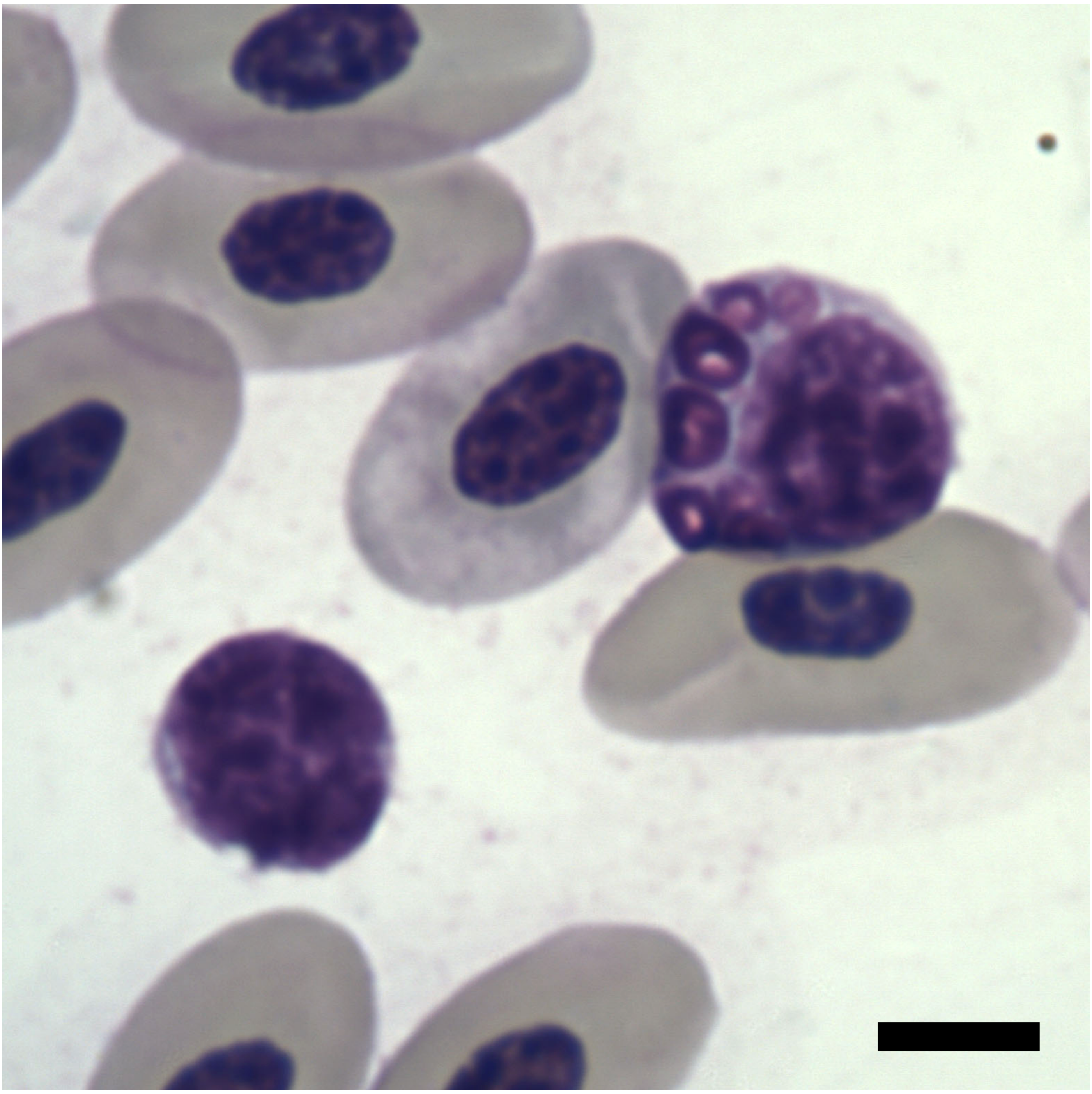

Supplement: Supplementary file 3 — Figure S1. Cytoplasmic inclusions in a lymphocyte of an African penguin (Spheniscus demersus) infected by “Candidatus Anaplasma sphenisci”. The lymphocyte with cytoplasmic inclusions (upper right) and a normal lymphocyte (lower left) are shown. Cytoplasmic inclusions had a width of 1.98 ± 0.54 μm (range = 0.86–3.10 μm). Modified Wright-Giemsa stain. Scale-bar: 5 μm. (PDF 8025 kb) [file 13071_2018_3089_MOESM3_ESM.pdf]
