# Supplementary material for: A novel candidate species of Anaplasma that infects avian erythrocytes
Source: Parasit Vectors. 2018 Sep 24;11:525. doi: 10.1186/s13071-018-3089-9 (PMC6154407; doi:10.1186/s13071-018-3089-9)

Additional file 4: Figure S2. Phylogenetic relationships of "*Candidatus* Anaplasma sphenisci" as determined by different phylogenetic methods based on partial sequences of the *16S rRNA* and *groEL* genes. Branch lengths are drawn proportionally to evolutionary distance (scale-bars are shown). Numbers adjacent to nodes indicate bootstrap values (a, b, d, e) or posterior probabilities (c, g).

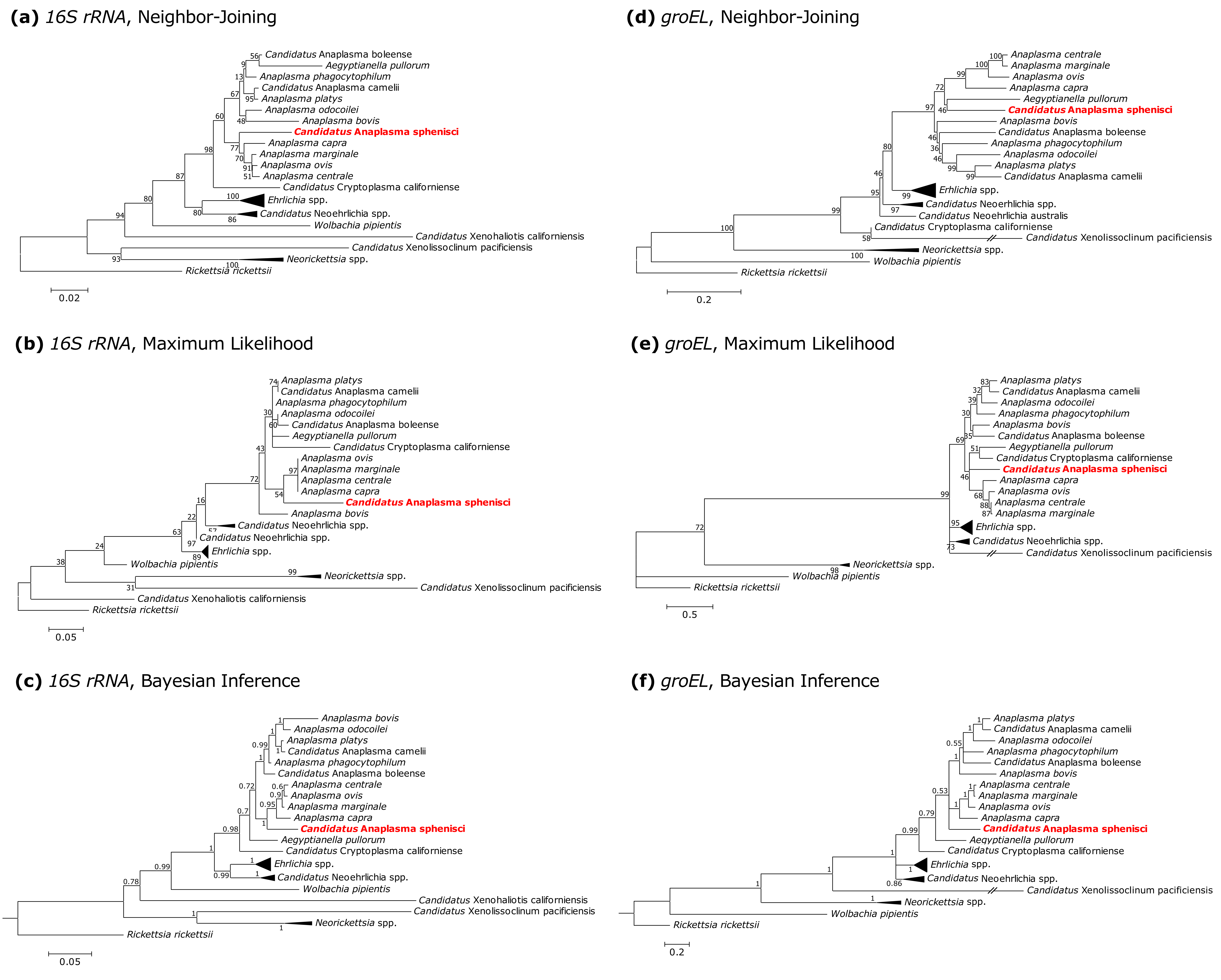

Supplement: Supplementary file 4 — Figure S2. Phylogenetic relationships of “Candidatus Anaplasma sphenisci” as determined by different phylogenetic methods based on partial sequences of the 16S rRNA and groEL genes. Branch lengths are drawn proportionally to evolutionary distance (scale-bars are shown). Numbers adjacent to nodes indicate bootstrap values (a, b, d, e) or posterior probabilities (c, g). (PDF 110 kb) [file 13071_2018_3089_MOESM4_ESM.pdf]
